# Familial Hypercholesterolemia: Do HDL Play a Role?

**DOI:** 10.3390/biomedicines9070810

**Published:** 2021-07-13

**Authors:** Juan Pedro-Botet, Elisenda Climent, David Benaiges

**Affiliations:** 1 Endocrinology and Nutrition Department, Hospital del Mar, 08003 Barcelona, Spain; 61063@parcdesalutmar.cat (E.C.); 96002@parcdesalutmar.cat (D.B.); 2 Department of Medicine, Universitat Autònoma de Barcelona, Campus Universitari Mar, 08003 Barcelona, Spain; 3 Institut Hospital del Mar d’Investigacions Mèdiques (IMIM), 08003 Barcelona, Spain

**Keywords:** familial hypercholesterolemia, cardiovascular disease, high density lipoproteins, HDL functionality, genetics, epigenetics

## Abstract

Cardiovascular disease (CVD) in heterozygous familial hypercholesterolemia (HeFH), the most frequent monogenic disorder of human metabolism, is largely driven by low-density lipoprotein (LDL) cholesterol concentrations. Since the CVD rate differs considerably in this population, beyond the lifetime LDL cholesterol vascular accumulation, other classical risk factors are involved in the high cardiovascular risk of HeFH. Among other lipoprotein disturbances, alterations in the phenotype and functionality of high-density lipoproteins (HDL) have been described in HeFH patients, contributing to the presence and severity of CVD. In fact, HDL are the first defensive barrier against the burden of high LDL cholesterol levels owing to their contribution to reverse cholesterol transport as well as their antioxidant and anti-inflammatory properties, among others. In this context, the present narrative review aimed to focus on quantitative and qualitative abnormalities in HDL particles in HeFH, encompassing metabolic, genetic and epigenetic aspects.

## 1. Introduction

Heterozygous familial hypercholesterolemia (HeFH), the most frequent human metabolism monogenic disorder caused by mutations in the genes encoding for the low-density lipoprotein (LDL) receptor [1], apolipoprotein (Apo) B-100 [2], proprotein convertase subtilisin/kexin-type 9 (PCSK9) [3] or Apo E [4], entails high LDL cholesterol concentrations, resulting in a high lifetime risk for cardiovascular disease (CVD). In this respect, a subject with a pathogenic FH mutation and LDL cholesterol > 190 mg/dL has a 22-fold increased risk of premature CVD compared with a mutation-negative subject with LDL cholesterol < 130 mg/dL [5]. Fortunately, the introduction and widespread use of statins have markedly improved the prognosis of HeFH patients owing to their efficacy in LDL cholesterol reduction [6,7,8,9].

Although CVD in HeFH is largely driven by LDL cholesterol concentrations, its prevalence is extremely variable, even in subjects sharing the same pathogenic mutation [10]. Besides specific HeFH risk factors such as the type of mutation or the presence of tendon xanthomas [11], classical risk factors such as age, male sex, smoking, overweight/obesity, hypertension and low high-density lipoprotein (HDL) cholesterol may also account for the CVD rate differences in the HeFH population [9,12,13,14,15,16].

**Figure 1:**
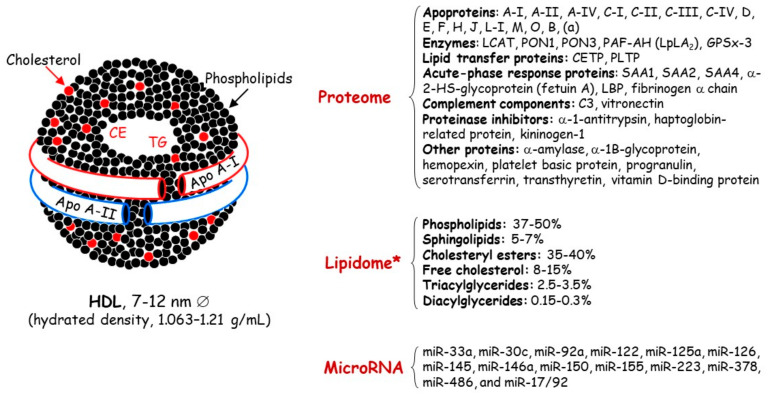
Main lipid and protein components of high-density lipoproteins. Apo, apolipoprotein; CE, cholesteryl esters; C3, complement 3; CETP, cholesteryl ester transfer protein; GSPx-3, glutathione selenoperoxidase 3; HDL, high-density lipoproteins; LCAT, lecithin:cholesterol acyltransferase; LBP, lipid-binding serum glycoproteins; Lp-PLA2, lipoprotein-associated phospholipase A2; MiRNA, microRNA; PAF-AH, platelet-activating factor acetyl hydrolase; PLPT, phospholipid transfer protein; PON, paraoxonase; SAA, serum amyloid A; TG, triglycerides. * HDL content in mol% of total lipids.

**Figure 2:**
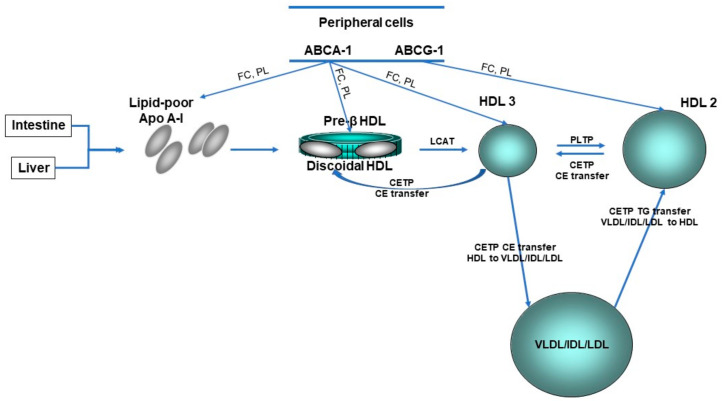
HDL lifecycle. The liver and gut synthesize apolipoprotein (Apo) A-I which become discoid nascent HDL after interaction with the ATP-binding cassette transporter A-1 (ABCA-1). Pre-β HDL is converted by lipidation with free cholesterol (FC) and phospholipids (PL) via ABCA-1 and ABCG-1 and cholesterol esterification via lecithin:cholesterol acyltranferase (LCAT) to spherical HDL3. HDL3 is converted to HDL2 by the fusion of small HDL particles activated by phospholipid transfer protein (PLTP). CE, cholesteryl ester; CETP, cholesteryl ester transfer protein; HDL, high-density lipoprotein; IDL, intermediate-density lipoprotein; LDL, low-density lipoprotein; TG, triglyceride; VLDL, very low-density lipoprotein.

Concerning HDL, these nanoparticles are traditionally characterized by their cholesterol and protein content (Figure 1); however, this cholesterol is not directly involved in the atheroprotective functions of HDL [17,18]. Discovered in 1929 by Macheboeuf [19] at the Pasteur Institute in Paris as lipid-rich α-globulins isolated from horse serum, these lipoproteins exhibit heterogeneity in their structure, metabolism and biological functions (Figure 2). It must be taken into account that HDL particles, the first defensive barrier against the burden of high LDL cholesterol levels, are subject to constant intravascular remodeling that generates multiple HDL phenotypes. The present narrative review aimed to focus on quantitative and qualitative abnormalities in HDL particles in HeFH, encompassing metabolic, genetic and epigenetic aspects.

## 2. Low HDL Cholesterol Phenotype in HeFH

Since the Framingham Heart Study in 1986 [20], the epidemiological association between low HDL cholesterol levels and an increased risk of coronary heart disease has been well established in several large population cohorts [21,22]. Based on these data, low HDL cholesterol concentrations are <1.0 mmol/L (40 mg/dL) for men and <1.2 mmol/L (48 mg/dL) for women.

One of the first references in the scientific literature stemmed from a letter by Seftel [23] to the Editor of the New England Journal of Medicine pointing out from Goldstein et al. data [24] the low HDL cholesterol levels in FH subjects. With all subsequent clinical studies taken together, ambivalent results regarding HDL cholesterol concentrations have been reported in this specific population. Low HDL cholesterol concentrations have been associated with a 37% relative increase in cardiovascular risk [12,25] and suggested as a strong marker of preclinical carotid atherosclerosis in HeFH patients [26]. Furthermore, from a French–Canadian cohort, Khoury et al. [27] discovered HeFH individuals aged > 70 years without cardiovascular events and tried to establish CVD-free survival factors. Beyond LDL cholesterol, female sex, non-smoking, high HDL cholesterol and hyperadiponectinemia were the main markers of longer CVD risk-free survival in HeFH. By contrast, no differences in HDL cholesterol concentrations were found in other studies when HeFH were compared with non-FH populations [28,29,30]. Therefore, environmental and genetic interactions involved in HDL metabolism could explain, at least in part, these discrepancies [17,18].

HDL cholesterol concentrations are influenced by the turnover of Apo A-I, their major protein component. Kinetic studies with stable isotopes have supported increased HDL-Apo A-I catabolism and secretion, thus maintaining its serum concentration balanced in HeFH patients [31]. The former finding may be related to enhanced cholesteryl ester transfer protein (CETP) activity in HeFH that leads to the triglyceride enrichment of HDL and/or to Apo E-enriched HDL that is catabolized by an Apo E receptor-mediated pathway; this concurs with the smaller HDL particle size found in HeFH patients [32]. Additionally, FH patients have elevated concentrations of small nascent preβ1-HDL particles [33,34], but lower levels of large HDL2 particles compared to normolipidemic subjects [33]. Interestingly, elevations in plasma apo E have been reported in FH patients [35], and the APOE genotype might influence plasma HDL cholesterol levels in these patients [36]. Another Apo differentially expressed in HeFH is Apo L1, with a decreased content of this protein in HDL3 particles [37]. A specific analysis of the HDL3 subfraction in that study revealed a coordinated decrease in the content of Apo L1 and lecithin:cholesterol acyltransferase (LCAT), a key enzyme in HDL metabolism, in HeFH patients with a fatal myocardial infarction during follow-up.

## 3. Dysfunctional HDL Particles in HeFH

**Figure 3:**
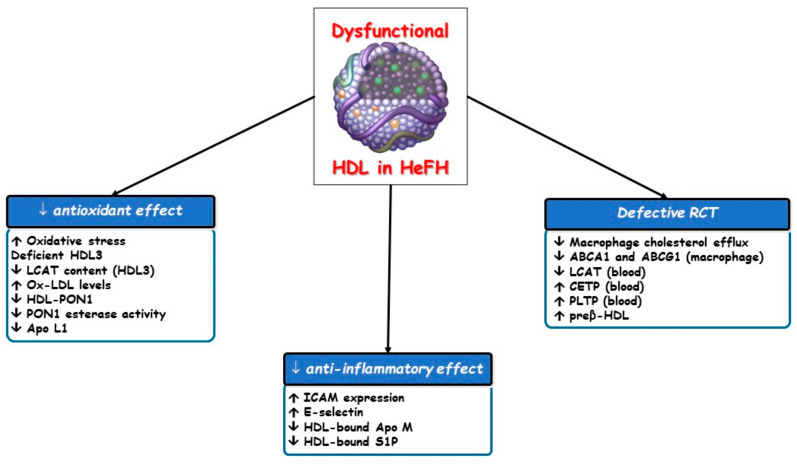
Impaired effects of dysfunctional HDL particles in HeFH. ABCA1, ATP-binding cassette transporter A1; ABCG1, ATP-binding cassette transporter G1; Apo, apolipoprotein; CETP, cholesteryl ester transfer protein; HDL, high-density lipoprotein; ICAM, intracellular adhesion molecule; LCAT, lecithin: cholesterol acyltransferase; Lp-PLA2, lipoprotein-associated phospholipase A2; PLTP, phospholipid transfer protein; PON1, paraoxonase type 1; RCT, reverse cholesterol transport; S1P, sphingosine-1-phosphate.

HDL functions are often impaired in different clinical settings including FH [17,38,39,40] owing to compositional changes in proteins, enzymes, lipids and microRNA which entail the loss of their atheroprotective properties (Figure 3).

### 3.1. Defective Reverse Cholesterol Transport (RCT)

Since peripheral tissue is unable to excrete excess free cholesterol, which is toxic for cells, it must be removed by RCT to the liver for utilization and excretion with bile, a role of HDL. Most of the cholesterol is effluxed from cholesterol-laden macrophages to nascent or mature HDL particles and finally transported to the liver in both esterified and unesterified forms. In primary cardiovascular prevention in the general population, cholesterol efflux capacity and the total HDL particle number are each inversely associated with cardiovascular events, regardless of the HDL cholesterol concentration and of each other [22,41]. Hypercholesterolemia induces lipidomic and proteomic variations in HDL particles, thereby damaging their ability to promote macrophage cholesterol efflux. This finding stemmed from a study on ischemia-reperfusion injury in pigs, in which the beneficial vascular effects of HDL were abolished by hypercholesterolemia [42].

**Figure 4:**
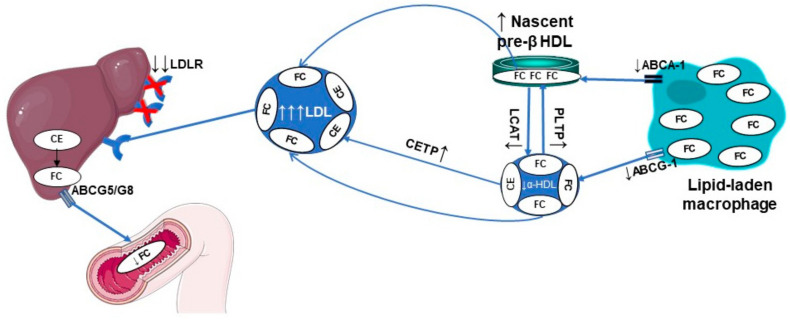
Defective macrophage reverse cholesterol transport in HeFH. ABCA1, ATP-binding cassette transporter A1; ABCG1, ATP-binding cassette transporter G1; ABCG5/G8, ATP-binding cassette transporter G5/G8; Apo, apolipoprotein; CE, cholesteryl ester; CETP, cholesteryl ester transfer protein; FC, free cholesterol; HDL, high-density lipoprotein; LCAT, lecithin: cholesterol acyltransferase; LDL, low-density lipoprotein; LDLR, low-density lipoprotein.

Notable evidence points to an altered macrophage-specific RCT in HeHF [43] (Figure 4). An impaired RCT, specifically related to the HDL2 subclass, with an inverse relationship between HDL efflux capacity and the development of atherosclerosis has been described in homozygous and HeFH patients [30]. Nevertheless, those patients were on high-intensity hypocholesterolemic therapy including LDL apheresis and RCT was assessed ex vivo. The decreased efflux of cellular cholesterol to HDL was confirmed when HDL particles were isolated in a study conducted in 71 HeFH patients and 66 normolipidemic subjects, leading to a reduced esterification by LCAT which was associated with an increased CVD risk [44]. Thus, the centripetal transport of cholesterol from the peripheral cells to feces appears altered in HeFH.

Versmissen et al. [45] showed that plasma from HeFH patients without CVD had a higher cholesterol efflux capacity than plasma from those with CVD, in comparison to their respective non-FH counterparts. This higher efflux capacity may involve differences in HDL composition including cholesterol, sphingosine-1-phosphate (S1P) and Apo M concentrations. In fact, increased S1P levels in HDL might also point to differences in HDL functionality other than cholesterol efflux capacity. Thus, the cardiovascular risk in HeFH patients appears to be modulated by factors not related to the LDL receptor locus that modifies the HDL composition and the effectiveness of RCT.

More recently, Cedó et al. [46] contributed to the clarification of the role of LDL in macrophage cholesterol efflux. Firstly, they reported that the LDL receptor regulates the reverse transport of macrophage-derived unesterified cholesterol via the concerted action of the HDL–LDL axis in hypercholesterolemic mice. They then assessed the HDL-associated remodeling of lipid transfer proteins and enzymes and their potential to alter HDL composition and macrophage cholesterol efflux in non-treated HeFH patients with an identified LDLR mutation and in normolipidemic subjects of a similar age and sex [34]. Among the most outstanding results, a high LDL particle concentration was linked to dysfunctional HDL particles characterized by their altered remodeling and an impaired capacity to promote cholesterol removal from macrophages. The higher CETP-facilitated lipid transfer, along with the reduced LCAT activity in adolescents with HeFH, indicates that these functional abnormalities may be primarily responsible for the altered HDL remodeling and functionality in younger patients with HeFH.

Regarding other changes in HDL composition, a depletion in phospholipids and an increased relative content of sphingomyelin can impair the capacity to sustain cellular cholesterol efflux in HeFH subjects [33,38]. On the other hand, in severe HeFH, LDL apheresis was highly efficient in lowering not only LDL cholesterol concentrations but also large ApoE-containing HDL, pre-β HDL, plasma CETP activity and cellular cholesterol efflux measured ex vivo [47], thus reflecting a transient defective RCT.

### 3.2. Other Altered HDL Atheroprotective Effects

Several lines of evidence have suggested enhanced chronic low-grade inflammation and systemic oxidative stress as well as impaired antioxidant and anti-inflammatory effects of HDL in HeFH [39,48]. Specifically, a diminished ability to inhibit intracellular adhesion molecule (ICAM)-1 expression in human umbilical vein endothelial cells after tumor necrosis factor-alpha (TNF-α) induction was demonstrated in HDL3 of HeFH patients with a body mass index > 25 kg/m^2^ and premature CVD [49]. In addition, raised soluble ICAM-1 concentrations in HeFH patients compared with non-FH controls were also reported. Moreover, HDL particles of FH patients were shown to be less potent in reducing the repletion of pro-inflammatory oxidized lipids in LDL compared with particles isolated from normolipidemic subjects [50]. Thus, a modified HDL3 anti-inflammatory function would less effectively neutralize the inflammatory scenario caused by the high LDL cholesterol levels in HeFH patients.

Although HDL particles are minor contributors to the overall antioxidant capacity of plasma [51], we must not forget that HDL3 particles are those with the most potent ability to protect LDL from oxidative damage [18], and this antioxidant property has been mainly attributed to LCAT [52]. Thus, a decreased LCAT content in HDL3 particles could reduce their antioxidant potential and favor LDL oxidation and atherosclerosis progression, thereby significantly increasing cardiovascular risk in FH patients. In fact, HeFH patients with corneal arcus, an independent predictor of CVD [53], have a lower content of Apo L1, LCAT and paraoxonase type 1 (PON1) in HDL particles [39].

PON1 is an HDL-related enzyme capable of hydrolyzing different substrates which include oxidative pro-oxidant species [54]. In HeFH patients, no association was found between peripheral concentrations of PON1 and carotid intima-media thickness, levels of oxidized LDL and the high sensitivity C-reactive protein [55]. More recently, PON1 esterase activity was found to be reduced in HeFH patients compared to healthy relatives [56]. These results should be considered preliminary and thus interpreted with caution.

On the other hand, the type and content of phospholipids are important regulators of HDL functions, producing an interesting relationship between the phosphatidylcholine/sphingomyelin ratio and the antioxidant activity of the HDL3 fraction. Decreased surface total phosphatidylcholine content with raised sphingomyelin and saturated fatty acid contents have been reported in HDL particles isolated from FH patients; this may reflect an increased surface rigidity and an ensuing impaired antioxidant capacity of HDL in FH [50].

### 3.3. MicroRNA Transport

MicroRNA are important regulators of a large variety of biological processes including lipoprotein metabolism [57]. Desgagné et al. [58] characterized the whole HDL microtranscriptome by next generation sequencing technology in healthy subjects and showed that HDL carry a microRNA profile distinct from that of other carriers in plasma which is not sex-dependent. The transport of endogenous microRNA by HDL was first proposed by Vickers et al. [59] who isolated and distinguished the HDL–microRNA profile in healthy volunteers and HeFH patients, noting significant differences. In this respect, hsa-miR-223, hsa-miR-105 and hsa-miR-106a were observed in the HDL of HeFH patients. In an explorative study, D’Agostino et al. [60] evaluated the association between miR-33a/b and miR-200c and their positive correlation in the plasma of HeFH children and found that they were upregulated. Since then, increasing attention has been paid to achieving a better understanding of the link between HDL–microRNA and HeFH, and HDL-miR-486 and HDL-miR-92a levels found to be more expressed in the LDLR-null group than the LDLR-defective group [61].

## 4. Genetics and Epigenetics

In a recent Mendelian randomization study, Prats-Uribe et al. [62] demonstrated that some qualitative HDL traits related to their size, particle distribution and cholesterol and triglyceride content were associated with cardiovascular risk, while HDL cholesterol concentrations were not. This relationship could be mediated by several HDL-related proteins which could be proposed as potential therapeutic targets in cardiovascular prevention.

As it could not be otherwise, most genetic background studies in HeFH aimed to determine the genetic polymorphisms responsible for high LDL cholesterol levels. Although low HDL cholesterol concentrations have been shown to affect the severity of the clinical phenotype, little is known about the genetic determinants that may explain a large part of HDL cholesterol variation in the HeFH population. In the largest exploratory Dutch study, van Aalst-Cohen et al. [63] selected 25 DNA polymorphisms in nine genes involved in HDL cholesterol. As expected, the effect of individual genetic polymorphisms on HDL cholesterol concentration was not exciting. In particular, the CETPTaqIB polymorphism demonstrated the strongest association with HDL cholesterol levels but accounted only for 1.1% of this variation. However, in combination with sex and environmental factors, the combined effects of polymorphisms involved in HDL metabolism could have explained up to 32.5% of the variation in HDL cholesterol levels.

Epigenetics refers to the heritable and reversible regulation of gene transcription by molecular mechanisms regardless of the DNA sequence. Previous epigenetic studies indicated that DNA methylation, the most understood and stable epigenetic mark, could explain the missing heritability of most complex traits, including plasma lipid levels [64]. In this context, epigenetic changes may account for lipoprotein profile variability, CVD and FH phenotype variability [32,65,66]. Langmann et al. [67] found that epigenetic modifications within the ATP-binding cassette transporter A1 (ABCA1) gene promoter contributed to the interindividual variability in plasma HDL cholesterol levels in FH patients. Next, Guay et al. reported that DNA methylation levels at the ABCA1, CETP and lipoprotein lipase (LPL) gene promoter loci were particularly associated with variations in HDL size and cholesterol content, as well as a history of CVD in HeFH [68,69]. Later, they described genome-wide epigenetic perturbations which are associated with interindividual HDL cholesterol level variability in HeFH [70]. More specifically, they asserted that DNA methylation variability at multiple loci may explain a significant percentage of the missing HDL cholesterol heritability. They also identified the TNNT1 gene as a new candidate gene for HDL particle phenotype variability. They then demonstrated that both TNNT1 DNA methylation levels and the TNNT1 c.-20G>A polymorphism were associated with HDL cholesterol concentrations in FH and non-FH subjects, and with the prevalence of CVD in FH and non-FH men [71]. These results suggest that both epigenetic and genetic modifications at the 19q13.42 locus are associated with low HDL cholesterol levels and cardiovascular risk. Furthermore, they observed that a higher phospholipid transfer protein (PLTP) DNA methylation was associated with smaller HDL particles and lower concentrations of HDL-phospholipid and HDL cholesterol in men with HeFH. Additionally, they detected that higher hepatic lipase (LIPC) DNA methylation was associated with a lower HDL particle size, HDL-phospholipid, HDL cholesterol and fasting triglyceride concentrations in men [72].

## 5. Impact of Current FH Lipid-Lowering Drugs on HDL Functionality

Although cardiovascular risk reduction in FH patients is clearly linked to the decline in LDL cholesterol levels by statins, these drugs have been shown to improve or restore the functionality of some components of dysfunctional HDL [73,74,75]. In this respect, statin therapy increases PON1 activity and concentrations in HeFH patients [75].

PCSK9 inhibitors have represented a substantial change in the clinical management of hypercholesterolemia, mainly in the FH population [76,77], due to their high lipid-lowering efficacy and their preventive effects in CVD [78,79]. PCSK9 is involved in cholesterol homeostasis through the LDL receptor degradation pathway. Circulating PCSK9 has been shown to bind to LDL, lipoprotein(a) and HDL [80]. PCSK9 gain-of-function mutations are associated with increased HDL cholesterol and Apo A-I levels [81]. Furthermore, epidemiological studies such as the Dallas Heart Study [82] and the Quebec Child and Adolescent Health and Social Survey [83] also reported a positive relationship between PCSK9 and HDL cholesterol levels. The effect of PCSK9 on the LDL receptor and others including the ApoE receptor two and the very-low-density lipoprotein receptor appears to be responsible for this relationship, possibly by reducing the uptake of the ApoE-containing HDL [84].

However, PCSK9 inhibition by monoclonal antibodies, alirocumab and evolocumab, is associated with a significant increase in HDL cholesterol and apo A-I concentrations [85,86]. This beneficial effect could be explained at least in part by the LDL reduction as the cholesterol acceptor from HDL, an effect that overcomes the improvement of the LDL receptor-mediated ApoE-containing HDL [84]. Thus, the role of PCSK9 on HDL composition and subclasses as well as the impact on RCT should be investigated.

## 6. Conclusions

Although the hallmark of HeFH is the hypocatabolism of LDL particles, growing evidence has highlighted the existence of additional metabolic disturbances in other plasma lipoproteins. In this respect, HDL particles in HeFH patients display qualitative abnormalities, including compositional changes, a reduced capacity to promote cholesterol efflux from macrophages and impaired anti-inflammatory and antioxidant activities, thereby suggesting a potential role of dysfunctional HDL in the high cardiovascular risk of HeFH patients. Thus, although LDL cholesterol concentration is crucial for FH clinical management, further measuring of HDL cholesterol will, at best, only partially reflect the potential role of HDL in CVD risk assessment. This has led to interest in developing new HDL metrics such as particle number, average size, subclasses, and functional assays that might better indicate the atheroprotective functions of HDL [87]. Given the important contributions of epigenetic studies, further research is required to unravel the key molecular processes involved in the epigenetic control of HDL in both FH and non-FH populations.
